# Comparative analysis of short - term functional outcomes and quality of life in a prospective series of brachytherapy and Da Vinci robotic prostatectomy

**DOI:** 10.1590/S1677-5538.IBJU.2016.0098

**Published:** 2017

**Authors:** Cristina García-Sánchez, Ana A. Román Martín, J. Manuel Conde-Sánchez, C. Belén Congregado-Ruíz, Ignacio Osman-García, Rafael A. Medina-López

**Affiliations:** 1 Virgen del Rocío Universitary Hospital, Seville, Spain

**Keywords:** Prostatectomy, Quality of Life, Brachytherapy, Robotic Surgical Procedures

## Abstract

**Introduction:**

There is a growing interest in achieving higher survival rates with the lowest morbidity in localized prostate cancer (PC) treatment. Consequently, minimally invasive techniques such as low-dose rate brachytherapy (BT) and robotic-assisted prostatectomy (RALP) have been developed and improved. Comparative analysis of functional outcomes and quality of life in a prospective series of 51BT and 42Da Vinci prostatectomies DV

**Materials and Methods:**

Comparative analysis of functional outcomes and quality of life in a prospective series of 93 patients with low-risk localized PC diagnosed in 2011. 51patients underwent low-dose rate BT and the other 42 patients RALP. IIEF to assess erectile function, ICIQ to evaluate continence and SF36 test to quality of life wee employed.

**Results:**

ICIQ at the first revision shows significant differences which favour the BT group, 79% present with continence or mild incontinence, whereas in the DV group 45% show these positive results. Differences disappear after 6 months, with 45 patients (89%) presenting with continence or mild incontinence in the BT group vs. 30 (71%) in the DV group. 65% of patients are potent in the first revision following BT and 39% following DV. Such differences are not significant and cannot be observed after 6 months. No significant differences were found in the comparative analysis of quality of life.

**Conclusions:**

ICIQ after surgery shows significant differences in favour of BT, which disappear after 6 months. Both procedures have a serious impact on erectile function, being even greater in the DV group. Differences between groups disappear after 6 months.

## INTRODUCTION

Prostate cancer (PC) is the most common non-cutaneous cancer detected in males in the Western world (1). Retropubic radical prostatectomy has been the treatment of choice for localized PC in patients with a life expectancy ≥10yrs. Nowadays, the growing interest in achieving higher survival rates with lower morbidity has led to the development and rise of minimally invasive techniques, such as low-dose rate BT and robotic-assisted prostatectomy (RALP) (2).

A variety of therapies can be used to treat low-risk PC, according to D’Amico classification (3); BT and RALP are two of them. Nevertheless, the use of one technique or another depends on the consensus between physician and patient. Current systematic studies on the management of localized PC conclude that all the treatments affect functional outcomes and quality of life with varying degrees, severity and duration. But, so far, there is not enough evidence to support one clinical procedure over the other.

The objective of the present study is to compare functional outcomes and quality of life in a prospective series of 51BT and 42Da Vinci robotic prostatectomies (DV) performed in our institution, being to this date the only report comparing both techniques, currently at their peak.

## MATERIALS AND METHODS

From January through December 2011, 93 males diagnosed with low-risk localized PC in our institution chose BT or Da Vinci prostatectomy treatment. The choice was a personal decision once patients had been orally informed about the different therapies and after they had filled up a Validated Tool for Decision-making (4), which is a simple document explaining the different therapies for PC and side effects. Once patients had read the document and solved any doubts, 51 chose low-dose rate BT and 42DV prostatectomy.

Low-dose rate BT consists in the permanent implantation of Rapid Strand Iodine-125 seeds at a dose of 145Gy. Transperineal implantation of the seeds is performed in lithotomy position guided by transrectal echography, performing planimetry and previous dosimetry in the same procedure (real-time scheduling).

Robotic prostatectomy was carried out through laparoscopy using 3 ports in an inverted-U configuration of the robot arms (left ilioinguinal access port, left and right pararectal ports), a supraumbilical port for the optical trocar, a right secondary ilioinguinal port (12mm) and an optional right pararectal port (5mm). We performed antegrade dissection with neurovascular bundle preservation.

Both procedures were carried out by the same team of 4 urologists with wide experience.

The inclusion criteria for both techniques were strictly observed: clinical staging T1-T2a, Gleason score <7, PSA level <10, Body max index <35, prostate volume <50cc.

In our prospective series, we compared functional outcomes and quality of life before and after surgery during the first follow-up year. At months 3, 6, 9 and 12 patients filled up ICIQ (International Consultation on Incontinence Questionnaire) (5), IIFE (International Index of Erectile Function) (6) and the short-form SF36 test (physical and mental) (7) validated for Spanish. Through the ICIQ we evaluated urinary incontinence as mild (1-7), moderate (8-12) and severe (13-21) and with the IIEF score, erectile dysfunction was rated as severe (<15), moderate (15-20) or mild (21-25).

For the descriptive analysis, qualitative variables were expressed as absolute and relative frequencies and quantitative variables as median and interquartile range, when the distribution was normal or mean and standard deviation, when the distribution was not normal (Kolmogorov-Smirnov test). Chi square test was used to compare qualitative variables and Student T test or Mann Whitney U test for quantitative variables. A value of p <0.05 was considered as statistically significant. SPSS v20 was used to perform the statistical study.

## RESULTS

Mean age of patients in the BT group was 64yrs vs. 60yrs in the DV group, being this difference significant (p <0.05). The groups under study were homogeneous and no statistically significant differences were observed in regard to tumour staging, PSA level at diagnosis, Gleason score or preoperative IPSS. We found differences in the surgical removed piece volume, preoperative maximum flow and SF36 (physical) (Table-1).

**Table 1:** Pre-treatment description of age, PSA level, Gleason score, IPSS, maximum flow and SF36 in the two groups of patients.

Pre- Intervention	BT	DV	p
Age (mean)	64	60	*
PSA	5.8	6.3	NS
Gleason	6	6	NS
IPSS	6	6	NS
Maximum flow	22	15	*
SF 36 (physical)	50	53	*
Prostate volume	31	39	*

**NS =** No significant; * = <0.05

Most prostatectomy specimens were pT2c (51%), 27% of pT3 were understaged (24% pT3a and 3% pT3b) and 9% had positive surgical margins (predominantly unifocal). No death occurred in the mean follow-up period of 8 months (4-10).

The comparison of pre-and postoperative data revealed differences in IPSS and maximum flow which worsen significantly in the BT group (Table-2).

**Table 2:** Comparison of pre and post-treatment outcomes in the BT and DV groups.

	BT	p	DV	p
IPSS (pre/post)	6 / 14	*	6 / 7	NS
Maximum flow (pre/post)	22 / 16	*	15 / 16	NS
Hemoglobin (pre/post)	15 / 14	*	15 / 13	*
ICIQ < 7 (pre/post)	83%/79%	NS	79%/45%	*
IIEF > 21 (pre/post)	60%/24	*	45%/10%	*
SF 36 (Pre/post)	105/ 105	NS	103/ 101	NS

**Pre/Post =** before intervention / 1 month after intervention; **NS =** No significant; * = <0.05

**Figure 1:**
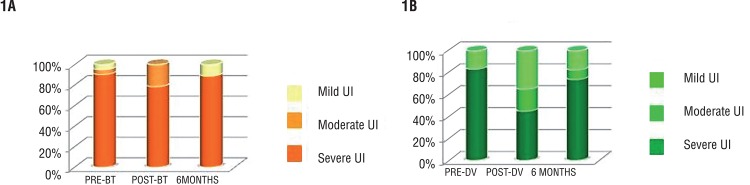
Percentage of mild, moderate or severe incontinence pre-treatment, at 3 months after treatment and from month 6 onwards.

ICIQ at the first revision shows significant differences in favour of the BT group, as 40 patients (79%) were continent or present with mild incontinence, whereas only 19 patients in the DV group showed such positive results. Differences disappear after 6 months, when we found the same percentage of continent patients in both groups, 89% (45 patients) in BT and 71% (30 patients) in DV group (Table-2 and Figure-1).

**Figure 2:**
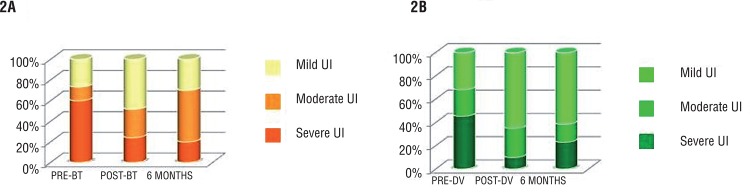
Percentage of mild, moderate or severe erectile dysfunction pre-treatment, 3-6 months after treatment and from month 6 onwards.

Both techniques have a serious impact on erectile function, with significant worsening of postoperative IIEF, being even more significant in the DV group (Table-2). Differences disappear after 6 months (65% with IIEF >15 in the BT group vs. 39% in the DV group) (Figure-2).

No significant differences were observed when we compared quality of life before surgery and in the different revisions (at months 3, 6 and 9) (Table-2).

In regard to further variables that could affect quality of life, hospital stay was significantly longer in the DV group (3 vs. 1 days) as well as postoperative pain, assessed by the Visual Analog Scale (VAS) (2 vs. 1) and mean indwelling catheter time (16 vs. 1 days).

We observed a significant decrease of pre-and postoperative hemoglobin levels in both series, being greater in the DV group (Table-2). Although no differences are found in the number of patients showing complications (4 in each group) Table-3, the severity of complications, according to Clavien-Dindo classification, varies, with 2 patients in the DV group (48%) requiring conversion to open surgery.

**Table 3:** Post-treatment complications.

Procedure	Complication	Clavien-Dindo	Approach
BT	AUR	II	Conservative
	AUR	II	Conservative
	UI	I	Medical
	UI	I	Medical
DV	Bleeding	III	Reconversion
	Difficult dissection	III	Reconversion
	Bleeding	II	Transfusion
	Urinary leak	II	Conservative

**AUR =** Acute Urinary Retention; **UI =** Urinary Infection.

## DISCUSSION

Prostate cancer (PC) is the most common non-cutaneous cancer affecting males in the Western world. Since the prostate-specific antigen (PSA) screening became widely used for the early detection of PC in the early 90s, cancer-specific mortality has changed drastically (8). As an increasing number of tumours detected are localized, now the main objective of physicians is to improve morbidity (incontinence and erectile dysfunction) while maintaining the control of the disease (9). Thus, minimally invasive techniques have emerged in recent years to treat low-risk PC (BT, cryotherapy, HIFU, robotic prostatectomy). All of them try to provide positive oncological outcomes and improve the functional outcomes of radical prostatectomy. Recently, different series of patients undergoing low-dose rate BT or robotic-assisted prostatectomy have been reported with promising outcomes (10-13).

Both low-dose rate BT and robotic-assisted prostatectomy are equally recommended to treat PC in low-risk patients (according to D’Amico classification) with PSA levels <10ng/mL, Gleason score ≤6 and tumour staging T1-T2a. Nowadays, the final decision to undergo one procedure or another depends on the consensus between physician and patient. In our series, 27% of the radical prostatectomies were pT3, similar to other series (11).

Among the advantages of BT we must mention that it is a short, minimally invasive procedure (45-90 minutes) which does not require prolonged hospital stay. Also, BT delivers radiation in the prostate gland minimizing the radiation dose to surrounding healthy tissues. This would diminish radiation-related side effects such as erectile dysfunction or urinary incontinence, thus improving patients’ quality of life. The improvement of functional outcomes with the use of robotic-assisted prostatectomy is due to the greater precision and technical skill achieved with the use of articulated instruments, ergonomic manipulation and 3D vision with 10x magnification.

A systematic review of reports comparing prostatectomy and BT to treat organ-confined PC concluded that BT shows similar outcomes to other therapies used for this type of tumours, at least in low-risk patients (14).

Nevertheless, none of the two procedures lack shortcomings or complications. Although the learning curve of robotic prostatectomy seems to be faster and this procedure provides ergonomic advantages for surgeons in comparison to conventional laparoscopy, the time required to prepare the Da Vinci system is twice the time necessary in conventional laparoscopy and the main drawback it presents is the loss of tactile sensation (15). On the other hand, according to some reports, BT shows a high incidence of local recurrence and complications (16).

Despite their limitations both procedures are two minimally invasive techniques which have proven useful to treat PC. However, there is no clear evidence to decide which of the two is the most adequate treatment for low-risk localized PC.

In our study, both the patients undergoing BT and those undergoing DV had similar PSA levels, Gleason score and tumour staging. The preoperative characteristics of the patients included in each group can be compared with those of the series so far published (9-13, 17-18). In our series, we found differences regarding mean age which can be explained by a tendency of ageing patients to choose BT to avoid the anesthetic complications of surgery. Age (64 vs. 60) is statistically different but we think it is not clinically relevant. SF36 and maximum flow could be clinically relevant, and because there is no randomization those differences can only be explained because people who understand that they are physically impaired prefer braquitherapy. Nevertheless, after treatment there is not differences in SF-36. Flowmetry and IPSS worsen significantly after BT (IPSS 6 and maximum flow 22 before intervention vs. IPSS 14 and maximum flow 16 after intervention). This is one of the possible side effects of BT, mainly due to a syndrome of mixed urinary incontinence (irritative symptoms provoked by acute cystitis and obstructive symptoms resulting from the local inflammation following radioactive seeds implantation) which can be partially controlled with the administration of alpha-blockers and anti-inflammatories.

Although DaVinci surgery is said to reduce hospital stay in comparison to open surgery, in the group of patients undergoing DaVinci prostatectomy included in our study, we observed an increase in hospital stay, surgical times and surgeon’s fatigue when compared with patients undergoing BT. Likewise, DV patients showed worse outcomes in VAS and had to use an indwelling catheter for a longer period of time.

The comparison of functional outcomes between the two groups is the main basis of our report. The ability to attain a firm enough erection for sexual intercourse depends on a variety of factors such as age, comorbidity (diabetes and peripheral vascular disease), psychological factors, habits and social factors. Also, we must consider the effect of the surgical technique or procedure employed. All this together with the multiple definitions of the terms sexual potency and erectile dysfunction and the use of different validated questionnaires makes it difficult to evaluate sexual function and to compare outcomes (9, 22). The same is true when we analyse urinary incontinence.

The percentage of potent patients following DV prostatectomy reaches 7-86% (23-32), which varies depending on the series, age of patients and unilateral or bilateral neurovascular bundle preservation. In the BT group, the percentage of potent patients ranges between 11 and 98% depending on previous sexual function, age, race and BMI (33).

Sexual function is seriously affected in both groups. Although no statistically significant differences are observed, impairment of sexual function is greater in the DV group (IIEF >15 in 65% of patients in BT group vs. 39% in DV group). Differences disappear after the six month.

A high percentage of DV patients present with early recovery of urinary continence (91-96%) (12, 34, 35). Our BT group showed a higher continence in the revisions at months 3 and 6 (79% patients without incontinence or with mild incontinence in the BT group vs. 45% in the DV group). Differences disappear after 6 months (71% patients without incontinence or mild incontinence in the DV group vs. 89% in BT group).

Bradley et al. in a report of 2004 compared radical prostatectomy, BT and BT combined with external radiotherapy and found no differences regarding quality of life. They observed worse initial functional outcomes in the radical prostatectomy group, which disappeared with time (36).

## CONCLUSIONS

In our study, both patients undergoing BT and robotic-assisted prostatectomy experience a significant impairment of urinary continence and erectile function, especially the latter. Although initial functional outcomes are slightly better in the BT group, after six months patients undergoing robotic-assisted prostatectomy attain similar results to the BT group. No differences are observed in relation to quality of life when we compare both therapies.

To this date, there is no clear evidence to favour one technique over the other in relation to functional outcomes. Further studies comparing both techniques are necessary as well as the use of standardized validated questionnaires (IIEF, ICIQ, SF-36) before and after treatment and the objective quantification of urinary leaks (pad test) instead of the use of subjective quantification parameters.
